# Bisphosphonates and Dental Implants: A Systematic Review and Meta-Analysis

**DOI:** 10.3390/ma16186078

**Published:** 2023-09-05

**Authors:** Nabaa Sulaiman, Fadi Fadhul, Bruno Ramos Chrcanovic

**Affiliations:** 1 Faculty of Odontology, Malmö University, 214 21 Malmö, Sweden; nabaa.sulaiman@gmail.com (N.S.); fadi_970428@hotmail.com (F.F.); 2 Department of Prosthodontics, Faculty of Odontology, Malmö University, 214 21 Malmö, Sweden

**Keywords:** dental implants, bisphosphonates, implant failure, marginal bone loss, systematic review, meta-analysis

## Abstract

The purpose of the present systematic review was to investigate the influence of bisphosphonates (BPs) on the dental implant failure rate and marginal bone loss (MBL). An electronic search was undertaken in three databases, plus a manual search of journals. Meta-analyses were performed, besides a meta-regression in order to verify how the log odds ratio (OR) was associated with follow-up time. The five- and ten-year estimated implant survivals were calculated. The review included 33 publications. Altogether, there were 1727 and 21,986 implants placed in patients taking and not taking BPs, respectively. A pairwise meta-analysis (26 studies) showed that implants in BP patients had a higher failure risk in comparison to non-BP patients (OR 1.653, *p* = 0.047). There was an estimated decrease of 0.004 in log OR for every additional month of follow-up, although it was not significant (*p* = 0.259). The global estimated implant survival in patients taking BPs after 5 and 10 years was 94.2% (95% CI, 94.0–94.4) and 90.1% (95% CI, 89.8–90.3), respectively. It was not possible to make any reliable analysis concerning MBL, as only two studies reported MBL results separated by groups. There is a 65.3% higher risk of implant failure in patients taking BPs in comparison to patients not taking this class of drugs.

## 1. Introduction

Osteoporosis is a systematic skeletal disorder marked by low bone mass, microarchitectural deterioration of bone tissue and weakened bone strength, leading to an increased bone fragility with a higher risk for susceptibility to fracture risk [1]. Osteoporosis is one of the most prevalent bone disorders among the elderly in the world [2]. The greatest likelihood of developing osteoporosis is in Europe and North America as the average life expectancy of the population increases. Osteoporosis has become a major public health problem throughout the world, with approximately 200 million women being currently affected by the condition [3].

The condition of the bone determines what type of medication strategy is eligible, an anabolic one or an antiresorptive (AR) strategy. The aim of an anabolic strategy is to affect the amount of bone by stimulating the formation of new bone, while that of an AR strategy is to reduce the breakdown of old bone. The pharmacological treatment of osteoporosis has so far mainly focused on reducing the breakdown and preserving the existing bone, i.e., an AR strategy [4].

Bisphosphonates (BPs) comprise a group of AR drugs. BPs are analogs of pyrophosphates that are widely indicated to alter bone metabolism to prevent bone loss caused by ailments like osteoporosis, hypercalcemia of malignancy, Paget disease of the bone, and malignancies with metastasis to the bone [5,6]. In the case of osteoporosis, BPs improve the bones’ mechanical strength caused by an increase in bone mass, as well as by an improvement in bone architecture, thus reducing the risk of fractures [7].

However, BPs can also cause negative adverse effects, one of which being medication-related osteonecrosis of the jaws (MRONJ), defined as exposed necrotic bone with severe pain after invasive dental procedures with no history of craniofacial radiation therapy [6,8], which is associated not only with BPs, but also with other drugs, such as antiresorptive (denosumab) and antiangiogenic therapies [9]. Several hypotheses have been proposed in order to try to explain the fact that medication-related osteonecrosis has been showing a considerable occurrence in the jaws. These include an increased rate of bone remodeling in the jaws in comparison to other bones, and therefore, the jaws may be more predisposed to inhibition of osteoclastic bone resorption and of bone remodeling [10]. Moreover, there is also inhibition of angiogenesis [11], the possible influence of dental diseases and oral bacterial infections [12,13], compromised vitamin D functions in the realm of skeletal homeostasis and innate immunity [14], and constant local microtrauma [15], which may be the result of dental procedures such tooth extractions and/or the use of dentures [16,17], among other hypotheses [9].

By the year 2006, it was estimated that over 190 million oral BP prescriptions had been dispensed worldwide [18]. As the use of BPs seems to be high in the population, together with the fact that the prevalence of dental implant installation among adults with missing teeth has been showing a substantial increase in the last 20 years or so [19], it is important to know whether the modification of bone metabolism by BPs might be a risk factor for oral rehabilitation with dental implants.

The aim of the present review was, therefore, to test the null hypothesis of no difference in the implant failure rates and marginal bone loss (MBL) after the insertion of dental implants in patients taking BPs in comparison to patients not taking BPs against the alternative hypothesis of a difference, based on a systematic review of the literature.

## 2. Materials and Methods

The PRISMA Statement guidelines [20] were followed for this review. Registration in PROSPERO was undertaken (CRD42022342090).

### 2.1. Research Question

The PICO format was used to frame the research question: In patients (Participants) being rehabilitated with implant-supported dental prosthetic restorations (Intervention), is there a difference in the dental implant failure rate (Outcome) between patients taking or not taking BPs (Comparison)?

### 2.2. Search Strategies

Electronic searches were undertaken in October 2021, with complementary updated searches in October 2022, in three databases: PubMed/Medline, Web of Science (in “all databases”), and Science Direct. Time restrictions were not applied. The terms used were the following:

(“dental implant” OR “oral implant”) AND (bisphosphonate OR diphosphonate)

Related prosthodontic, implantology, maxillofacial, and specialist dental and oral journals (listed in the Supplementary Material) were manually searched. The reference list of the identified studies and the relevant reviews on the subject were also checked for possible additional studies. The grey literature was not searched.

### 2.3. Inclusion and Exclusion Criteria

Eligibility criteria included clinical studies with available data on implant failure in any group of patients taking BPs and being (or planned to be) rehabilitated with implant-supported dental prostheses. Only the cases rehabilitated with cylindrical screw-type modern dental implants of titanium (commercially pure titanium or titanium alloys) were considered. Case and technical reports, animal and in vitro studies, and reviews were excluded. Studies reporting cases rehabilitated with mini-implants, orthodontic, zygomatic, zirconia, subperiosteal, or hollow implants were also excluded.

### 2.4. Study Selection

The methodology has been described in the Supplementary Material.

### 2.5. Quality Assessment

The Quality Assessment Tool of the National Institutes of Health [21] was used. The methodology has been described in the Supplementary Material.

### 2.6. Definitions

An implant was considered a failure the patient presented signs and symptoms that led to implant removal. Implant failure could be either early (the inadequacy of the host to establish or promote osseointegration in the early stages of healing) or late (the failure of either the established osseointegration or function of dental implants) [22]. A fracture of an implant was also considered a failure [23].

The drugs considered as BPs included the following (in alphabetical order): alendronate, cimadronate, clodronate, etidronate, ibandronate, minodronate, neridronate, olpadronate, pamidronate, risedronate, tiludronate, and zoledronate [24].

An apical loss of marginal alveolar bone adjacent to the dental implant in relation to the marginal bone level detected initially after the implant was installed into the bone site was defined as MBL [25]. Studies were considered for inclusion in the review if the long-cone parallel technique for periapical radiographs had been used.

### 2.7. Data Extraction

The methodology has been described in the Supplementary Material.

### 2.8. Analyses

Meta-analysis was attempted between groups of patients (taking BPs or not). Implant failure was the dichotomous outcome measure evaluated. Weighted mean differences were used to construct forest plots of marginal bone loss, a continuous outcome. The statistical unit for “implant failure” and “marginal bone loss” was the implant. Whenever the outcomes of interest were not clearly stated, the data were not used for analysis. The I^2^ statistic was used to express the percentage of the total variation across studies due to heterogeneity, with 25% corresponding to low heterogeneity, 50% to moderate, and 75% to high. The inverse variance method was used for random-effects or fixed-effects models. Where statistically significant (*p* < 0.10) heterogeneity was detected, a random-effects model was used to assess the significance of the treatment effects. Where no statistically significant heterogeneity was found, an analysis was performed using a fixed-effects model [26]. The estimates of relative effect for dichotomous outcomes were expressed in odds ratio (OR) and in mean difference (MD) in millimeters for continuous outcomes, both with a 95% confidence interval (95% CI). Only if there were studies with similar comparisons reporting the same outcome measures was meta-analysis to be attempted. A funnel plot (plot of effect size versus standard error) was planned to be drawn if more than 10 studies could be included in the meta-analysis. Asymmetry of the funnel plot may indicate publication bias and other biases related to sample size, although the asymmetry may also represent a true relationship between the trial size and effect size.

The event rates for implant failure in patients taking BPs were calculated for each study by dividing the total number of events by the total exposure time in years, according to what has been described elsewhere [27]. The 5- and 10-year survival proportions were calculated via the relationship between the event rate and survival function S, S(T) = exp (−T*event rate), assuming constant event rates. Calculation of the global estimated survival after 5 and 10 years was performed with the random-effects DerSimonian–Laird method [28].

The OpenMeta[Analyst] software [29] was used to analyze the data. The funnel plot was generated with the software OpenMEE [30].

## 3. Results

### 3.1. Literature Search

**Figure 1:**
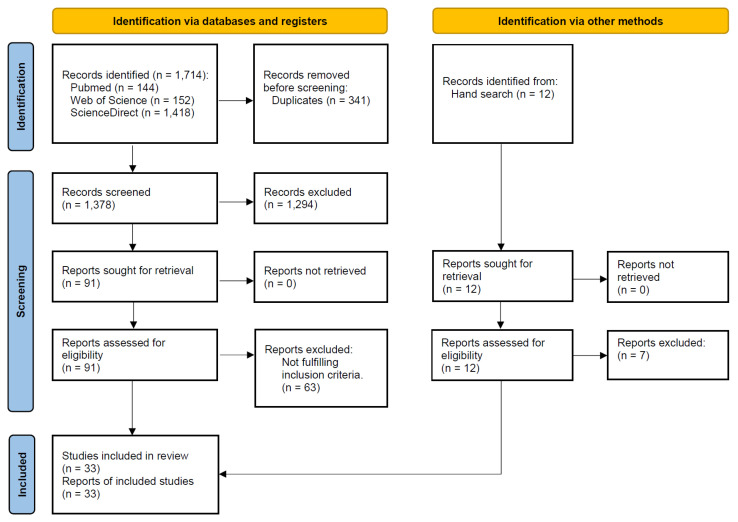
Study screening process.

The study selection process is summarized in Figure 1. The search strategy in the databases resulted in 1714 papers, of which 33 publications were included in the review.

### 3.2. Description of the Studies

Table S1 (see Supplementary Material) shows the detailed data of the 33 included studies [31,32,33,34,35,36,37,38,39,40,41,42,43,44,45,46,47,48,49,50,51,52,53,54,55,56,57,58,59,60,61,62,63]. The articles were published between 2006 and 2022. Twenty-six studies were unicenter and six were multicenter. Seven studies were prospective studies and 26 were retrospective studies. The institution where the study was carried out was one or more universities for twenty-two studies, a private dental practice for ten studies, and the public service for two studies. Different types of institutions could be included in multicenter studies. The studies were most commonly carried out in the USA, in 16 studies. Other common places for the studies were Italy in five cases, Sweden in two cases, and Austria, Canada, Germany, India, Iran, Japan, Portugal, Slovakia, South Korea, and Switzerland with one study each.

The mean follow-up ± standard deviation of 27 studies was 57.2 ± 55.2 months (min–max, 3–291). No clear time of follow-up nor the mean follow-up time was available for the other six studies.

Immediate prosthetic loading was applied in ten studies, early loading in one study, and delayed loading in seventeen studies. These loading protocols (either immediate, early, or delayed) could be applied either separately for all implants of a study or in combination for different implants of the same study. Information about the loading protocol was not available in 13 studies.

Most studies (*n* = 27) included implants installed in the maxilla and mandible; six studies included patients that received implants only in the mandibles, and one only in the maxillae.

Smokers were included in twenty studies, while in four studies there were no smokers among the patients, with no information available in nine studies.

Considering only the studies (*n* = 26) that had two groups (patients taking and patients not taking BPs) and that reported information on implant failure, there were 1727 implants that were placed in patients taking BPs and 21,986 implants placed in patients not taking BPs, and 64 and 786 implant failures in these groups, respectively. Six studies investigated the clinical outcomes of dental implants only in patients taking BPs, and 19 failures were observed in 1672 implants when these six studies are considered together. The implants most commonly used were from Nobel Biocare (Göteborg, Sweden) and Straumann (Basel, Switzerland). For 11 studies, there was no available information on which implant brand and/or system was used.

Eight studies reported results on MBL, of which only two [52,60] reported MBL results separated between the groups.

### 3.3. Quality Assessment

All studies were categorized as “good” by the quality assessment tool (Table S2—see Supplementary Material for detailed information). The issues most often identified in the studies were a lack of a good description of the statistical methods applied and treatment of patients that were not consecutively recruited to the study.

### 3.4. Meta-Analyses

**Figure 2:**
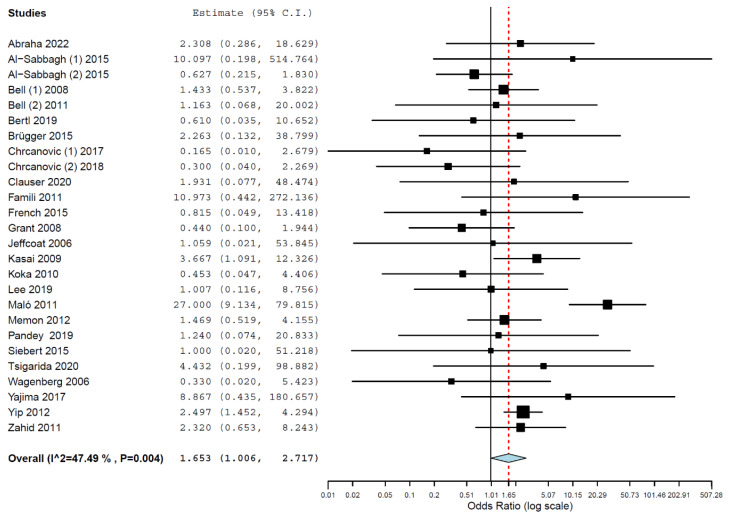
Forest plot for the event “implant failure”.

Implants placed in patients taking BPs had higher odds of failure than implants placed in patients not taking this class of drug (OR 1.653, 95% CI, 1.006, 2.717, *p* = 0.047; Figure 2). An OR of 1.653 means that failures of implants placed in patients taking BPs present a 1.653 higher risk of happening than failures of implants placed in patients not taking BPs; i.e., implants in patients taking BPs have a higher risk of failure by 65.3% in relation to implants in patients not taking BPs. A random-effects model was used for the pairwise meta-analysis due to heterogeneity (τ^2^ = 0.604, Chi^2^ = 47.613, I^2^ = 47.493, *p* = 0.004).

A meta-analysis comparing MBL between groups would not generate reliable results, due to only two studies reporting MBL separately by patients taking or not taking BPs. The result would be the same as the one already reported in a previous review [64], namely, a mean difference of 0.05 mm (95% CI, −0.12, −0.22; *p* = 0.590).

### 3.5. Meta-Regression

Of the studies that had two groups (patients taking and patients not taking BPs), twenty-two provided clear information about the follow-up time or mean follow-up time. No precise follow-up time information was available for the other four studies. These studies either provided this information as a period of years over which the patients received the implants and the subsequent time period when the study data were extracted [36,42,48], or conducted a survival analysis with no information about the mean follow-up time [51].

**Figure 3:**
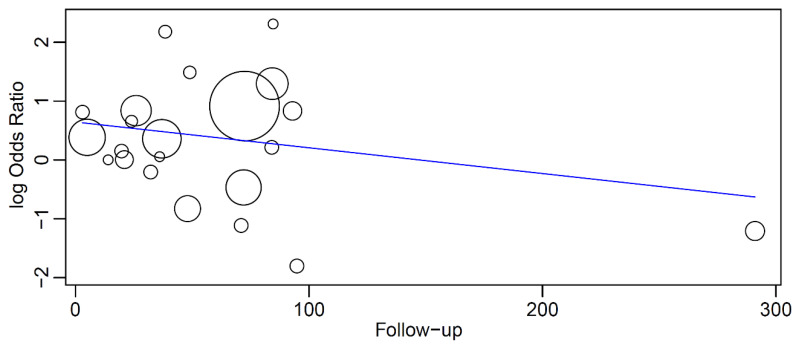
Relationship between the log odds ratio (OR) of implant failure between patients taking BPs and patients not taking BPs and the follow-up time (in months). Every circle represents a study and the size of the circle represents the weight of the study in the analysis. The regression line is represented by the blue line.

In a meta-regression with these 22 studies, having the follow-up as covariate in relation to OR, it was observed that the OR decreased with an increase in the follow-up time, although without significance (*p* = 0.259) (Figure 3). The linear regression equation of this meta-regression was

y = 0.645 − 0.004x

where:

Intercept = 0.645 (0.049, 1.240), standard error 0.304, *p* = 0.034
Follow-up = −0.004 (−0.012, 0.003), standard error 0.004, *p* = 0.259

### 3.6. Estimated Survival

The global estimated implant survival in patients taking BPs, taking together the 26 studies that provided precise information on follow-up, after 5 and 10 years was 94.2% (95% CI, 94.0, 94.4; standard error 0.1, *p* < 0.01; heterogeneity: I^2^ = 99.95, *p* < 0.01) and 90.1% (95% CI, 89.8, 90.3; standard error 0.1, *p* < 0.01; heterogeneity: I^2^ = 99.97, *p* < 0.01), respectively.

### 3.7. Publication Bias

**Figure 4:**
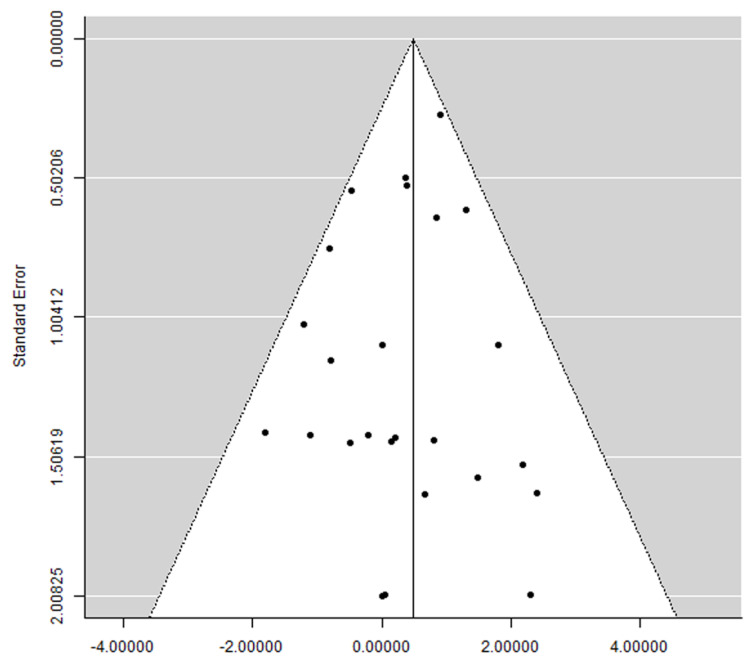
Funnel plot.

A clear asymmetry was not observed in the funnel plot (Figure 4), suggesting the absence of publication bias.

## 4. Discussion

The main finding of the present review was that patients taking BPs presented a statistically significant higher risk of dental implant failure than patients not taking this class of drug. The null hypothesis was therefore rejected. The hypothesis could not be verified concerning MBL, as only one of the included studies reported MBL results separated between the groups of patients.

The finding of a higher risk of implant failures in patients taking BPs is probably mainly related to some potential negative effects of BPs.

BPs’ ability to prevent bone resorption by inhibiting osteoclast activity inhibits bone remodeling and turnover and this may prevent the repair of microdamage and impair the osseointegration of dental implants in patients taking this medicine [65]. The results of an in vitro and in vivo study suggested that BP treatment inhibits angiogenesis by a significant dose-dependent decrease in endothelial cell proliferation and inhibition of capillary formation [66]. The final outcome from a poorly vascularized bone can be a greater risk of the absence of functional cells, such as osteoblasts, since it is the furthest from the blood supply. This fact, associated with the suppression of bone turnover by BP and in parallel with the effect of occlusal forces, reduces the mechanical properties of bone and may affect the implant survival rate. Sites with a poor bone quality and lack of bone volume may negatively affect the implant failure rates [67].

The drug administration method is an important issue. Higher potencies and doses of BPs are typically administered intravascularly (IV), which results in a greater potential for side effects (including multiple forms of nephrotoxicity, osteonecrosis of the jaw, hypocalcemia, and flu-like symptoms) [68] and in a significantly faster accumulation of BP in bone in comparison to oral agents [69]. BP loads bone and accumulates in bone 142.8 times faster when administered intravenously in comparison to when administered orally [70]. Therefore, higher implant failure rates than the ones reported here can be expected when IV administration is used, as there is no information about the use of IV BP in the included studies of the present review.

The period that patients have been under BP therapy may also play an important role in the survival of implants. The biological plausibility of this finding rests on the fact that patients treated with IV BPs or long-term use of oral BPs are at greatest risk to develop MRONJ [71], a condition that hypothetically influences the rate of implant failure negatively. The present results showed that the OR of an implant failure in BP patients in relation to non-BP patients decreased with an increased follow-up time, although without a mathematical significance. The lack of significance could be related to the lack of long-term studies, as only one study with information on failures and on follow-up time followed the patients for longer than 100 months.

The higher risk of implant failure may also be influenced by the patients’ compliance to the BP therapy. The evidence suggests that patients with multiple chronic conditions have poor compliance and persistence with oral BP medications, because they may take the drug incorrectly (dosage and time) or not at all [72]. On the other hand, IV BP therapy and monthly infusions ensures full compliance by reason of increased perceived convenience than daily oral therapy [73].

Last but not least, the different degrees of potency among different BPs is another point of significance, and therefore different BPs may have different potentials to affect bone metabolism and cause adverse effects [74] that could directly or indirectly have an effect on dental implant survival.

The results of the present review suggest a similar relationship between BPs and higher implant failure rates as a previous review published in 2016 [64], although with data from more studies. The previous review included 18 studies, resulting in a statistically significant negative effect size for patients taking BPs, but stated that it was not possible to clearly conclude that BPs may be a negative factor affecting dental implant survival due to limited data. The present review adds more evidence to the matter, although most of the studies still present low specificity to the issue, and therefore appropriately designed studies are still needed in order to confirm this cause–effect relationship.

### Limitations of the Present Systematic Review

Many confounding factors may have had some impact on the clinical outcomes and not only the ingestion or not of BPs by the patients. There was a considerable number of confounding factors, and there was no information about how many implants were inserted and failed in several different conditions for most (if not all) of the studies. For example, many studies reported the presence of smokers among the patients, as well as diabetic patients, patients submitted to chemotherapy, patients taking selective serotonin reuptake inhibitors or proton-pump inhibitors, and implants placed in fresh extraction sockets, factors that may have a considerable impact on implant failure rates. Individuals may have more than one local and/or systemic risk factor [75], and it is difficult to estimate the effect of these factors on the survival rate if these are not identified separately between the different implant groups.

Second, the retrospective nature of many studies results in flaws manifested by the gaps in information.

Third, most studies presented small cohort sizes and short follow-ups.

Fourth, the great majority of the studies included in the review are marked by low specificity, namely, the assessment of the possible effect of BPs on the clinical outcomes of the dental implants was not the central focus of the investigation.

## 5. Conclusions

The overall evidence from the present systematic review suggests that patients taking BPs present a higher risk of implant failure than patients not taking BPs. This should be considered in treatment planning and management of implant patients.

## Data Availability

The data presented in this study are available within the article and Supplementary Material.

## Supplementary Materials

The following supporting information can be downloaded at: https://www.mdpi.com/article/10.3390/ma16186078/s1, Table S1: Detailed data of the included studies, Table S2: Quality Assessment Tool according to the National Institutes of Health (NIH).
