# Supplementary material for: Bisphosphonates and Dental Implants: A Systematic Review and Meta-Analysis
Source: Materials (Basel). 2023 Sep 5;16(18):6078. doi: 10.3390/ma16186078 (PMC10532755; doi:10.3390/ma16186078)
Supplement: Supplementary file 1 [file materials-16-06078-s001.zip › materials-2586091-supplementary.pdf]

**SUPPLEMENTARY MATERIAL**

a. Dental implant-related journals included in the manual search;
b. Study selection;
c. Quality assessment;
d. Data extraction;
e. Table S1. Detailed data of the included studies;
f. Table S2. Quality assessment of the included studies, according to the National Institutes of Health (NIH).

## a. Dental implant-related journals included in the manual search

Clinical Implant Dentistry and Related Research, Clinical Oral Implants Research, European Journal of Oral Implantology, Implant Dentistry, International Journal of Implant Dentistry, International Journal of Oral and Maxillofacial Implants, International Journal of Oral Implantology, International Journal of Prosthodontics, Journal of Clinical Periodontology, Journal of Oral Implantology, Journal of Periodontology, Journal of Prosthetic Dentistry, Journal of Prosthodontics, Journal of Prosthodontic Research.

## b. Study selection

The titles and abstracts of all reports identified through the electronic searches were screened independently by two reviewers (FF, NS). For studies appearing to meet the inclusion criteria, or for which there were insufficient data in the title and abstract to make a clear decision, the full report was obtained. The full text assessment was carried out independently by two reviewers. Any disagreements were solved by discussion and if needed by a third reviewer (BRC).

## c. Quality assessment

The NIH quality assessment tool calculates the study quality on the basis of nine criteria. The ratings on the different items were used by the reviewers to assess the risk of bias in the study due to flaws in study design or implementation. The studies were classified as "good," "fair," or "poor" quality. In general terms, a "good" study has the least risk of bias, and results are considered to be valid. A "fair" study is susceptible to some bias deemed not sufficient to invalidate its results. The fair quality category is likely to be broad, so studies with this rating will vary in their strengths and weaknesses. A "poor" rating indicates significant risk of bias. Studies of "good" quality were judged to have at least 7 points.

## d. Data extraction

From the studies included in the final analysis, the following data were extracted (when available): year of publication, study design and setting, number of patients, patients' age, BP treatment details, implant healing period, implants used (model and brand), number of failed and placed implants, implant surface modification, jaws receiving implants (maxilla and/or mandible), type of prosthetic rehabilitation, opposing dentition, marginal bone loss, presence of smokers, and follow-up time. When needed, authors were contacted for additional information when data were missing.

**Table S1. Detailed data of the included studies.**

| Study | Year | Study Design | Country / Setting | Patients (male/female; bisphosphonate) (n) | Patients' Age Range (mean) (years) | Healing period / loading | Bisphosphonate therapy | Observations |
|---|---|---|---|---|---|---|---|---|
| Abraha | 2022 | RA (multicenter) | Germany / Hospital + University | 106 (25/81; 3) | NM (58.4) | Delayed (3 mo) | NM | 12 smokers, 5 diabetic, all patients submitted to Le Fort I and interpositional grafting |
| Al-Sabbagh (1) | 2015 | RA (unicenter) | USA / University | 203 (83/120; 20) | 21-90 (55.5) | NM | History of oral bisphosphonate therapy for 3 years or more | No cases of osteonecrosis |
| Al-Sabbagh (2) | 2015 | RA (unicenter) | USA / University | 415 (174/241; 39) | NM (59.4) | NM | NM | 46 smokers, 43 diabetic, 59 osteoporosis |
| Bell | 2008 | RA (unicenter) | USA / Private practice | NM (NM; 42) | NM | NM | Alendronate (34 patients), Risedronate (6 patients), Ibandronate (2 patients) | Grafting procedures in 30 patients of G1. Smokers were also included, but the exact number was not informed. No cases of osteonecrosis |
| Bell | 2011 | RA (unicenter) | USA / Private practice | 655 (NM) | NM | Delayed (3 mo) | NM | All implants placed in fresh extraction sockets, 83 implants in diabetic patients, 123 implants in smokers, no cases of osteonecrosis |
| Bertl | 2019 | RA (unicenter) | Austria / University | 444 (192/252; 31) | 65-91 (72) | NM | NM | 48 smokers, 47 diabetic, 41 osteoporosis, 384 patients with history of periodontitis, 48 taking selective serotonin reuptake inhibitors, 66 taking proton-pump inhibitors |
| Brügger | 2015 | RA (unicenter) | Switzerland / University | 1568 (776/792; 23) | 17-92 (53.6) | Delayed (4-14 wk) | Alendronate, ibandronate, pamidronate, risedronate, zoledronate | 190 light smokers<br>81 heavy smokers<br>38 diabetic<br>23 osteoporosis |
| Chrcanovic | 2017 | RA (unicenter) | Sweden / Public service | 999 (479/520; NM) | 14-90 (60) | Immediate, delayed | NM | Several systemic conditions identified among patients<br>263 light, heavy and extra-heavy smokers<br>23 former smokers |
| Chrcanovic | 2018 | RA (unicenter) | Sweden / Public service | 227 (95/132; NM) | NM | Immediate, delayed | NM | Several systemic conditions identified among patients<br>Smokers not excluded |

| | | | | | | | | |
|---|---|---|---|---|---|---|---|---|
| Clauser | 2020 | PS (multicenter) | Italy / Private practice | 214 (92/122; 1) | 17-84 (48.3) | Immediate | NM | 24 heavy smokers<br>4 extra-heavy smokers |
| Demarosi | 2010 | PS (unicenter) | Italy / University | 21 (2/19; 21) | 56-86 (67.3) | Delayed | Alendronate (18 patients), clodronate (2 patients), risendronate (1 patient) | All patients with osteoporosis<br>Only non-smokers |
| Famili | 2011 | RA (unicenter) | USA / University | 120 (0/120; 22) | > 50 | NM | Alendronate (16 patients), Risedronate (4 patients), Ibandronate (2 patient). Duration of use: >6 months-1 year (6 patients), >1 year (9 patients), >5 years (5 patients), unknown (2 patients). | No cases of osteonecrosis |
| French | 2015 | RA (multicenter) | Canada / Private practice | 2060 (922/1138; 34) | 15-85 (50.6) | Immediate, early, delayed | NM | 29 smokers (>15 cig/d)<br>27 diabetic<br>39 bruxers<br>14 autoimmune disease |
| Fugazzotto | 2007 | RA (multicenter) | USA / Private practice | 61 (0/61; 61) | 51-83 (NM) | Delayed (6 wk) | Alendronate or Risedronate, 35 or 70 mg/week, mean 3.3 years | 39 implants inserted in fresh extraction sockets, GBR in some cases, no cases of osteonecrosis |
| Grant | 2008 | RA (unicenter) | USA / Private practice | 458 (0/458; 115) | > 40 (67.4) | NM | Alendronate, Risedronate, Ibandronate. Mean 38 months. Before implant: 89 patients. After implant: 26 patients | No cases of osteonecrosis, 2 diabetic patients |
| Jeffcoat | 2006 | CCT (unicenter) | USA / University | 50 (0/50; 25) | NM | NM | Mean 3 years | All patients with osteoporosis, 2 smokers, no cases of osteonecrosis |
| Kasai | 2009 | RA (unicenter) | USA / University | 51 (0/51; 11) | 52-73 (NM) | NM | Alendronate | No smokers, no cases of osteonecrosis |
| Koka | 2010 | RA (unicenter) | USA / Private practice | 137 (0/137; 55) | > 50 | NM | NM | No cases of osteonecrosis |
| Lee | 2019 | RA (unicenter) | South Korea / University | 156 (70/86; NM) | 19-84 (59.9) | Delayed | NM | 18 diabetics, 21 osteoporosis, 3 chemotherapy |
| Leonida | 2012 | PS (unicenter) | Italy / University | 9 (1/8; 9) | 45-68 (NM) | Immediate | Risedronate (5 patients), Alendronate (4 patients), for less than 3 years. Suspension of | All patients had residual teeth with severe periodontal disease, 19 implants in fresh extraction sockets, no cases of osteonecrosis |

| | | | | | | | | |
|---|---|---|---|---|---|---|---|---|
| | | | | | | | bisphosphonate therapy 1 month after the surgery | |
| Maló | 2011 | RA (unicenter) | Portugal / Private practice | 245 (96/149; 4) | 23-85 (59) | Immediate | NM | 61 smokers<br>5 diabetic<br>6 autoimmune disease |
| Memon | 2012 | RA (unicenter) | USA / University | 200 (0/200; 100) | 47-90 (63) | Delayed (4-6 mo) | Risedronate (23 patients), Ibandronate (5 patients), Alendronate (72 patients). Duration of use: <1 year (20 patients), 1-3 years (19 patients), >3 years (15 patients), unknown (46 patients). | 8 smokers, 7 diabetic patients, grafting procedures in 88 implant sites, 60 nonsubmerged implants, no cases of osteonecrosis |
| Mozzati | 2015 | PS (unicenter) | Italy / University | 235 (0/235; 235) | 48-79 (60.7) | Immediate<br>Delayed | Oral BP: alendronate (141 patients), ibandronate (68 patients), risedronate (45 patients) | Sinus lift for 54 implants, no cases of osteonecrosis, 51 smokers, 21 diabetic patients |
| Pandey | 2019 | RA (unicenter) | India / University | 30 (0/30; 15) | NM (63) | NM | Alendronate 10 mg once daily | All patients with osteoporosis |
| Siebert | 2015 | PS (unicenter) | Slovakia / University | 24 (0/24; 12) | >54 | Delayed (6 wk) | Zoledronic acid (annual infusions of 5 mg) | Nonsmokers, no chemotherapy, no radiotherapy, no glucocorticosteroid therapy<br>12 osteoporosis |
| Shabestari | 2010 | RA (multicenter) | Iran / University + Private practice | 21 (0/21; 21) | 42-79 (53) | Delayed (3-5 mo) | Alendronate 35-70 mg/week, mean 20.5 months. Before implant: 7 patients. After implant: 14 patients | Grafting procedures in 5 patients, no cases of osteonecrosis |
| Tallarico | 2016 | PS (multicenter) | Italy / Private practice | 32 (0/32; 32) | 46-80 (64.6) | Delayed (3 mo) | Alendronate 70 mg/week or or 5–10 mg/day, for at least 3 years before implant placement | All implants in healed sites, no cases of osteonecrosis |
| Tsigarida | 2020 | RA (unicenter) | USA / University | 71 (41/30; 5) | NM (65) | Immediate<br>Delayed | NM | 6 smokers, 6 diabetic patients |
| Wagenberg | 2006 | RA (unicenter) | USA / University | 891 (NM) | 14-94 (57.9) | Immediate<br>Delayed (3-6 mo) | Alendronate | All implants placed in fresh extraction sockets, bone grafts were utilized in all cases in which there was a residual space around the implant, 13 implants in sinus- |

| | | | | | | | | |
|---|---|---|---|---|---|---|---|---|
| | | | | | | | | lifts, 323 implants in smokers, no cases of osteonecrosis |
| Wagenberg | 2013 | RA (unicenter) | USA / University | 541 (NM) | 12-88 (58) | Immediate Delayed | NM | All implants in fresh extraction sockets, 184 implants in smokers, no cases of osteonecrosis |
| Yajima | 2017 | RA (unicenter) | Japan / University | 25 (0/25; 11) | > 61 | NM | Alendronate | No diabetics, no smokers, no cases of osteonecrosis |
| Yip | 2012 | RA (unicenter) | USA / University | 337 (0/337; 20) | > 40 | NM | Alendronate, Risedronate | 52 smokers, 20 diabetic patients, no cases of osteonecrosis |
| Zahid | 2011 | RA (unicenter) | USA / University | 300 (NM; 26) | 17-87 (56) | NM | 35-70 mg/week, 6-192 months | Grafting procedures in 173 implant sites, 56 implants in smokers, no cases of osteonecrosis |

NM – not mentioned; PS – prospective study; CCT – controlled clinical trial; RA – retrospective analysis
wk – weeks; mo – months

**Table S2. Quality assessment of the included studies, according to the National Institutes of Health (NIH).**

| Study | Year | Was the study question or objective clearly stated? | Was the study population clearly and fully described, including a case definition? | Were the cases consecutive? | Were the subjects comparable? | Was the intervention clearly described? | Were the outcome measures clearly defined, valid, reliable, and implemented consistently across all study participants? | Was the length of follow-up adequate? [a] | Were the statistical methods well-described? | Were the results well-described? | Total (n/9) |
|---|---|---|---|---|---|---|---|---|---|---|---|
| Abraha | 2022 | 1 | 1 | 1 | 1 | 1 | 1 | 1 | 0 | 1 | 8/9 |
| Al-Sabbagh (1) | 2015 | 1 | 1 | 0 | 1 | 1 | 1 | 1 | 1 | 0 | 8/9 |
| Al-Sabbagh (2) | 2015 | 1 | 1 | 0 | 1 | 0 | 1 | 1 | 1 | 1 | 7/9 |
| Bell | 2008 | 1 | 1 | 1 | 1 | 0 | 1 | 1 | 0 | 1 | 7/9 |
| Bell | 2011 | 1 | 1 | 0 | 1 | 1 | 1 | 1 | 0 | 1 | 7/9 |
| Bertl | 2019 | 1 | 1 | 1 | 1 | 0 | 1 | 1 | 1 | 0 | 7/9 |
| Brügger | 2015 | 1 | 1 | 1 | 1 | 1 | 1 | 1 | 1 | 1 | 9/9 |
| Chrcanovic | 2017 | 1 | 1 | 1 | 1 | 1 | 1 | 1 | 1 | 1 | 9/9 |

| | | | | | | | | | | | |
|---|---|---|---|---|---|---|---|---|---|---|---|
| Chrcanovic | 2018 | 1 | 1 | 0 | 1 | 1 | 1 | 1 | 1 | 1 | 8/9 |
| Clauser | 2020 | 1 | 1 | 1 | 1 | 1 | 1 | 1 | 1 | 1 | 9/9 |
| Demarosi | 2010 | 1 | 1 | 1 | 1 | 1 | 1 | 1 | 1 | 1 | 9/9 |
| Famili | 2011 | 1 | 1 | 1 | 1 | 0 | 1 | 1 | 1 | 0 | 7/9 |
| French | 2015 | 1 | 1 | 0 | 1 | 1 | 1 | 1 | 1 | 1 | 8/9 |
| Fugazzotto | 2007 | 1 | 1 | 1 | 1 | 1 | 1 | 1 | 1 | 1 | 9/9 |
| Grant | 2007 | 1 | 1 | 1 | 1 | 0 | 1 | 1 | 0 | 1 | 7/9 |
| Jeffcoat | 2006 | 1 | 1 | 1 | 1 | 1 | 1 | 1 | 1 | 1 | 9/9 |
| Kasai | 2009 | 1 | 1 | 1 | 1 | 0 | 1 | 1 | 0 | 1 | 7/9 |
| Koka | 2010 | 1 | 1 | 0 | 1 | 1 | 1 | 1 | 0 | 1 | 7/9 |
| Lee | 2019 | 1 | 1 | 0 | 1 | 1 | 1 | 1 | 1 | 1 | 8/9 |
| Leonida | 2012 | 1 | 1 | 0 | 1 | 1 | 1 | 1 | 0 | 1 | 7/9 |
| Maló | 2011 | 1 | 1 | 1 | 1 | 1 | 1 | 1 | 0 | 1 | 8/9 |
| Memon | 2012 | 1 | 1 | 0 | 1 | 1 | 1 | 1 | 1 | 1 | 7/9 |
| Mozzati | 2015 | 1 | 1 | 1 | 1 | 1 | 1 | 1 | 1 | 1 | 9/9 |
| Pandey | 2019 | 1 | 1 | 0 | 1 | 1 | 1 | 1 | 0 | 1 | 7/9 |
| Shabestari | 2010 | 1 | 1 | 0 | 1 | 1 | 1 | 1 | 1 | 0 | 7/9 |
| Siebert | 2015 | 1 | 1 | 0 | 1 | 1 | 1 | 1 | 1 | 0 | 7/9 |
| Tallarico | 2016 | 1 | 1 | 1 | 1 | 1 | 1 | 1 | 1 | 1 | 9/9 |
| Tsigarida | 2020 | 1 | 1 | 0 | 1 | 1 | 1 | 1 | 1 | 1 | 9/9 |
| Wagenberg | 2006 | 1 | 1 | 1 | 1 | 1 | 1 | 1 | 0 | 1 | 8/9 |
| Wagenberg | 2013 | 1 | 1 | 1 | 1 | 1 | 1 | 1 | 0 | 1 | 8/9 |
| Yajima | 2017 | 1 | 1 | 0 | 1 | 1 | 1 | 1 | 1 | 1 | 8/9 |
| Yip | 2012 | 1 | 1 | 1 | 1 | 1 | 1 | 1 | 1 | 1 | 9/9 |
| Zahid | 2011 | 1 | 1 | 1 | 1 | 1 | 1 | 1 | 1 | 1 | 9/9 |

[a] 3 months of follow-up was chosen to be of adequate length.
